# Anonymous Pamphleteers

**Published:** 1843-04

**Authors:** 


### ANONYMOUS PAMPHLETEERS.

The Editor of the *Boston Medical and Surgical Journal* (March 15) speaks, in a tone of just indignation, of an anonymous pamphlet sent to him (from this city?) purporting to give "some account of the Faculty of the Louisville Medical Institute." He lashes its author or authors (for they are plural) to some purpose. Whether he has an inkling of whom he speaks, we do not know. He may rest assured, however, that he cannot say anything too severe of them. No epithets he could apply, would give any adequate idea of their cowardly meanness and malignity. Filthy and corrupt as they are, their sole gratification is in attempting to defile the good character of others. As with obscene birds, this occasional belching-forth of the putrid contents of their stomach is necessary we suppose to their own safety—the only other effect it has, is to make them a stench in the nostrils of all good men, and thus widen the circle around themselves. The publication of this pamphlet, cowardly and assassin-like as it is in the extreme, could scarcely add to their degradation, unless in that depth there might be a lower deep. The fact that they are ashamed to acknowledge their own filthy and leprous off-spring, might be taken as an evidence of some remnant of good in their depraved nature, if we did not know them to be as dastardly as they are dishonest, as bankrupt in moral courage as in all else.

The secret of this matter can be told in a very few words. This pamphlet is composed of a number of scurrilous articles which appeared in one of the papers of this city, soon after the nefarious scheme to transfer the Medical Institute was defeated. These articles were concocted by the same knavish triumvirate who were so deeply engaged in that scheme, and who participated so actively in the publication of the infamous report made to the City Council on the subject. (See this Journal for September and October, 1842.) Of course, men who were base enough to sanction and give currency (as in that Report) to statements which *they knew to be false*, would have no scruples about uttering stale and oft-repeated slanders, such as are contained in this precious pamphlet, with the addition of whatever their own malice and devilish ingenuity could invent. What villainous act they will next be caught in, remains to be seen. C.

